# TriBAFF-CAR-T cells eliminate B-cell malignancies with BAFFR-expression and CD19 antigen loss

**DOI:** 10.1186/s12935-021-01923-x

**Published:** 2021-04-17

**Authors:** Guangchao Li, Qing Zhang, Zhi Liu, Huijuan Shen, Yangmin Zhu, Zhao Zhou, Wen Ding, Siqi Han, Jie Zhou, Ruiming Ou, Min Luo, Shuang Liu

**Affiliations:** 1 Department of Hematology, Guangdong Second Provincial General Hospital, Guangzhou, Guangdong Province 510317 China; 2 Guangzhou Bio-gene Technology Co., Ltd, Guangzhou, Guangdong Province 510530 China; 3 Department of Medical Oncology, Jinling Hospital, Nanjing Clinical School of Southern Medical University, Nanjing, Jiangsu Province 210002 China; 4 Department of Hematology, People’s Hospital of Deyang City, Deyang, Sichuan Province 618000 China; 5 Department of Hematology, Guangdong Second Provincial General Hospital, Xin Gang Zhong Road 466#, Haizhu Distict, Guangzhou, Guangdong Province 510317 China

**Keywords:** BAFF, Trimer, CAR-T, Hematological tumor, Tumor relapse

## Abstract

**Background:**

To investigate the effect of TriBAFF-CAR-T cells on hematological tumor cells.

**Methods:**

TriBAFF-CAR-T and CD19-CAR-T cells were co-cultured with BAFFR-bearing B-cell malignancies at different effector/target ratios to evaluate the anti-tumor effects. *In vivo*, TriBAFF-CAR-T and CD19-CAR-T cells were intravenously injected into Raji-luciferase xenograft mice. CD19 antigens losing lymphoblasts was simulated by Raji knocking out CD19 (CD19^KO^) to investigate the effect of TriBAFF-CAR-T cells on CD19^KO^ Raji.

**Results:**

Both TriBAFF-CAR-T and CD19-CAR-T cells significantly induced the lysis of Raji, BALL-1, and Jeko-1. Moreover, when CD19-CAR-T cells specifically caused the lysis of K562 with overexpressed CD19, the lethal effect of TriBAFF-CAR-T cells was also specific for BAFFR-bearing K562 with increasing levels of interleukin-2 and INF-γ. The TriBAFF-CAR-T have the same effect with CD19-CAR-T cells in treating Raji xenofraft mice. TriBAFF-CAR-T cells also have great effect in CD19^KO^ Raji cells.

**Conclusions:**

In this study, we successfully constructed novel TriBAFF-CAR-T cells to eliminate BAFFR-bearing and CD19 antigen loss in hematological tumor cells.

## Background

Chimeric antigen receptor T (CAR-T) cells are an emerging and novel cell type that consist of an extracellular single-chain variable fragment (scFv) with a hinge region, a transmembrane domain, intracellular activation, and costimulation domains [1, 2]. The widely studied CAR-T cell targeting CD19 has shown excellent therapeutic efficacy against patients suffering from relapsed and refractory hematological malignancies (ALL) [3], relapsed or refractory large B cell lymphoma (LBCL) [4, 5], chronic lymphocytic leukemia [6]. At present, there are 4 CAR-T cell therapies were approved by the FDA, it is a promising strategy for cancer. However, CAR-T cells are often overactivated with the emission of significantly increased levels of serum inflammatory cytokines, which causes cytokine-release syndrome and other severe toxicities [7].The over expression of cytokine may be harmful to human health [8]. Moreover, the single target of CD19 is often accompanied by relapse and recurrence for the alternative dominant molecule or CD19 antigen loss in patients. Another explanation for recurrence after CD19-CAR-T cell therapy is the dysfunction or elimination of CAR-T cells [9, 10]. Therefore, it is necessary to identify a new target to treat patients who are CD19 negative, and to enhance the activation and persistence abilities of CAR-T cells. Comparisons between CD19, CD20, and CD22 CAR-T cell therapy have shown the efficacy of CD19-CAR-T cells, indicating the advantages of high-density antigens in B cells [11, 12]. To address the challenge of CD19 antigen loss, targets with high expression in B cells must be explored.

B cell activating factor (BAFF) is an essential member of the tumor necrosis factor superfamily that can be expressed on the surface of or secreted from B cells. BAFF plays a crucial role in mature B lymphocytes as well as on progenitor B cells, plasma cells, and even malignant B cells such as non-Hodgkin lymphoma [13–16]. BAFF is the ligand of three different receptors of BAFF receptor (BAFFR); transmembrane activator, calcium modulator, and cyclophilin ligand interactor (TACI) and B cell maturation antigen (BCMA) receptors, but the BAFFR only binds to BAFF [17–19]. BAFF-BAFFR interactions are critical for the maintenance, activation, and differentiation of B cells [20, 21]. Unlike BAFF, which is also expressed in T cells, dendritic cells, and other non-hematopoietic cells, BAFFR is a strict B-lineage marker [22, 23]. Although the alternation stages of malignant B cells are continuous in patients, the dedication of BAFFR expression on B lymphocytes limits the misses of progenitor cells, even the stem cell stage of B cells [24–26].

BAFF exists as a membrane-bound and soluble form. Unlike the membrane-bound form, soluble BAFF is often polymerized as trimers or oligomers [27]. However, the existence of soluble oligomer BAFF is currently under debate. Both forms of BAFF are biologically active [28]. So, BAFF may be a new target in CAR-T therapy for malignant B cells, it has the great significance when CD19 antigen loss after CAR-T treatment. After previously comparing the monomer and trimer BAFF (TriBAFF)-associated CAR-T cells, we found that TriBAFF directing CAR-T cell targeting BAFFR more efficiently inhibited tumor development both *in vitro* and *in vivo*.

In this study, we construed the TriBAFF-armed CAR-T cells and analysis the oncolytic effect of TriBAFF-CAR-T cells *in vitro* and *in vivo*. CD19^KO^ cells were used to measure the candidate effect of TriBAFF-CAR-T in CD19 antigen loss in patients after CD19-CAR-T treatment.

## Methods

### Cell lines and human blood samples

K562, Raji, BALL-1, and Jeko-1 cells were purchased from the American Type Culture Collection (ATCC) and grown in Dulbecco’s modified Eagle’s medium (DMEM) with 10 % fetal calf serum at 37 °C and 5 % CO_2_. Two patients with B-ALL from the Second People’s Hospital in Guangdong Province, China were enrolled in this study. Blood samples were obtained and PBMCs were separated from the blood using density gradient centrifugation. The procedures were approved by the independent ethics committee of the second People’s Hospital in Guangdong Province, China.

### Plasmids and recombinant proteins

Plasmids encoding Fc chimeric recombinant wild-type (WT) and mutated BAFF, WT, and mutated TriBAFF were designed and manufactured by artificial DNA synthesis. Moreover, these sequences were cloned into pcDNA3.1(+) vectors (Invitrogen). Plasmids were purified using the EndoFree Plasmid Giga Kit (#12,391; Qiagen) and were added to the culture of 293T cells for transfection. After four days, cultures were collected when cells reached a density of 4–5 × 10^6^ cells/mL. Cultures containing soluble recombinant protein were purified from conditioned media using protein A-sepharose beads (#6501-1; BioVision) and the nonspecific binding protein was removed according to the manufacturer’s instructions. The purified recombinant proteins were incubated with Raji cells to determine the interactions with the cell surface.

### CAR-T cell production

1. Construction of CAR DNA: CAR DNA was artificially synthesized by successful linkage of the BAFF or TriBAFF, signal peptide, hinge, and transmembrane region of CD8α, costimulatory domain of 4-1BB, and signal transduction domain of CD3ζ. The CAR cDNA and linear pCDH-EF1-MCS vector (a lentivector) were connected for 1 h at 22 ℃ to obtain the linked vector, which was transformed into Stbl3 *Bacillus coli* and verified using Sanger sequencing. The linkage system included 2 µL (50 ng) of linear pCDH-EF1-MCS vector, 10 µL (150 ng) of CAR, 1 µL of T4 DNA ligase, 2 µL of linkage buffer, and 5 µL of ddH_2_O.
2. Lentivirus package: The auxiliary plasmids of gag/pol, Rev, and VSV-G with the above lentivectors were added to DMEM for 15 min. The mixture was then added to 293T cells cultured at 37 °C and 5 % CO_2_ for 6 h. Then, 72 h after changing the fresh DMEM and adding 10 mM sodium butyrate, the lentivirus in the culture supernatant was harvested and purified.
3. T cell preparation: 30 mL of whole blood diluted with 30 mL of saline was slowly added to the surface of Ficoll solution. PBMCs were collected after density gradient centrifugation and washed with saline. Then, PBMCs were activated in X-VIVO culture medium (containing 50 ng/mL OKT3 and 300 IU/mL interleukin (IL)-2) for two days and expanded in 300 IU/mL X-VIVO. The 300 IU/mL of X-VIVO was replaced with a fresh equivalent every two days to maintain the cell concentration at (0.5 ~ 1) × 10^6^/mL.
4. Lentivirus transfection: A six-well plate was coated with 30 µg RetroNectin at 37 °C for 2 h. The coated plate was blocked with 2.5 % BSA in Hank’s solution at 37 °C for 0.5 h and washed with 2 % HEPES in Hank’s solution. The X-VIVO culture medium was added to the plate, the contents of which were then centrifuged for 2 h at 2000*g*. Then, 1 × 10^6^ T cells (CD3^+^% > 90 %) were added and centrifuged for 10 min at 1000*g*. The mixture of the cells co-cultured at 37 °C and 5 % CO_2_ for five days was finally used to detect the expression of BAFF CAR and TriBAFF CAR through anti-BAFF antibody, or CD19 CAR through human CD19-FITC (CD9-HF251, Acrobiosystems) using flow cytometry.

### Generation of overexpressed K562 cell lines

The full-length BAFF-R and CD19 genes were constructed into the lentivirus expression vector pCDH-EF1-MSC. The lentivirus was packaged and infected with K562 cells; then, puromycin (5 µg/mL) was used to obtain cell lines stably expressing K562-BAFFR and K562-CD19.

### Generation of stable CD19 knockout (CD19^KO^) cell lines

Stable CD19^KO^ cell lines were generated through the CD19-CRISPR-Cas9 technique, according to the manufacturer’s instructions for the LentiCRISPRv2 vector. Briefly, the sgRNA against CD19 (SEQ ID NO: 11: 5’-CAGTCCTATGAGGATATGAG-3) was cloned into the LentiCRISPRv2 vector, which was digested using BsmBI enzyme. The linkage system included 1 µL (50 ng) of linear LentiCRISPRv2 vector, 1 µL of double-stranded DNA corresponding to sgRNA, 1 µL of T4 DNA ligase, 5 µL of 2 × Quick linkage buffer, and 3 µL of ddH_2_O. Raji was then transfected with lentivirus packaged using LentiCRISPRv2-CD19-sgRNA, and the stably transfected Raji was then sorted with 5 µg/mL puromycin.

### Detection using flow cytometry

Cells were harvested as previously described and stained for 30 min at 4 °C with anti-BAFF-APC (#366,507; BioLegend), anti-BAFFR-APC (#316,916; BioLegend), and anti-CD19-FITC antibodies (#392,508; BioLegend). Cells were acquired and analyzed using flow cytometry (BD Biosciences).

### Cell proliferation assay

1 × 10^4^ Raji-luc cells were used, and the Raji cells stably expressing luciferase by a lentivirus gene delivery system were cultured in black 96-well plates (J09602, Jinan, Shanghai) with the recombinant WT and mutated BAFF or TriBAFF protein (5 µg/mL). After five days, Raji-luc cells were incubated with 100 µL of reconstituted reagent (Bright-Glo Luciferase Assay System, E2620) for 2 min to detect the fluorescence intensity (FLUOstar OMEGA). The fluorescence intensity of the negative control (NC; phosphate-buffered saline) was set as 100 %. Lastly, the cell proliferation of Raji-luc cells in each experimental group was evaluated.

### Cytotoxic CAR-T cell assay

CAR-T cells, the effector cells, were co-cultured with target cells (B cell lines, myeloid cell lines, or CD19^KO^ cell lines) at an effector:target cell ratio ranging from 1:1 to 1:10 for 12 h. The LDH release assay (ab102526; Abcam) was used to analyze the lysis of target cells while setting the cell lysate as the positive control (PC) and DMEM as the NC. Briefly, 100 µL of supernatant and 20 µL reaction solution were added to a 96-well plate in the dark, and the absorbance was measured at 590 nm after a 20–30 min reaction. Supernatants were sampled after coculturing for 12 h and an enzyme-linked immunosorbent assay was performed with human interferon gamma (IFN-γ; VAL104; R&D Systems) and an IL-2 kit (VAL110; R&D Systems), according to the manufacturer’s instructions.

The proportion of lysis or specific lysis (%) = [(absorbance of co-culture-NC background)-(auto-release LDH of the CAR-T-NC background)-(auto-release LDH of the target cells-NC background)]/[(maximum LDH release of target cells-volume correcting)-(auto-release LDH of target cells-NC background)] × 100 %.

### Mice and the B-ALL model

Four-week-old female B-NDG mice were purchased from Biocytogen, Beijing. Mice were housed in a pathogen-free animal facility with free access to pellet food (Keao, Beijing) and water at 21 ± 2 °C and were maintained in a 12 h light/dark cycle. All animal studies were performed in accordance with the guidelines of the People’s Republic of China’s Ministry of Health. Luciferase-expressing Raji-luc cells were injected intravenously into the B-NDG mice. To monitor tumor development, the mice were anesthetized with isoflurane for 5 min and intraperitoneally administrated D-luciferin (3 mg/mL) for 10 min before bioluminescence imaging. Indices of bioluminescence imaging included IrisSetting = “f/0.95,“ ExposureTime = “60000 ms,“ PreampGain = “4X,“ Binning = “1 × 1,“ Emission = “open,“ Excitation = “blocked.“ Images were analyzed using Milabs-OI-PP-v1.9 software.

### CAR-T cell therapy

The 15 B-NDG mice burdening of B-ALL were divided into three groups and treated with CAR-T or T cells once the tumor engraftment was confirmed using bioluminescence imaging (Day − 1). Mice in these groups were intravenously administered 2 × 10^6^ TriBAFF-CAR-T, CD19-CAR-T, or control T cells (without CAR), respectively. Imaging was performed on days 3, 7, and 14 to compare tumor development among groups. The survival rate of the mice was observed for up to 20 days.

### Statistical analysis

All statistical analyses were performed using SPSS 12.0. Data were analyzed using Student’s *t*-test or one-way analysis of variance between two or three groups. Survival data are reported in Kaplan-Meier plots and analyzed using log-rank tests. All data presented in figures are shown as means ± SD with three replicates.

## Results

### Mutated TriBAFF bind but could not activate BAFFR-bearing B cell lines

**Fig. 1:**
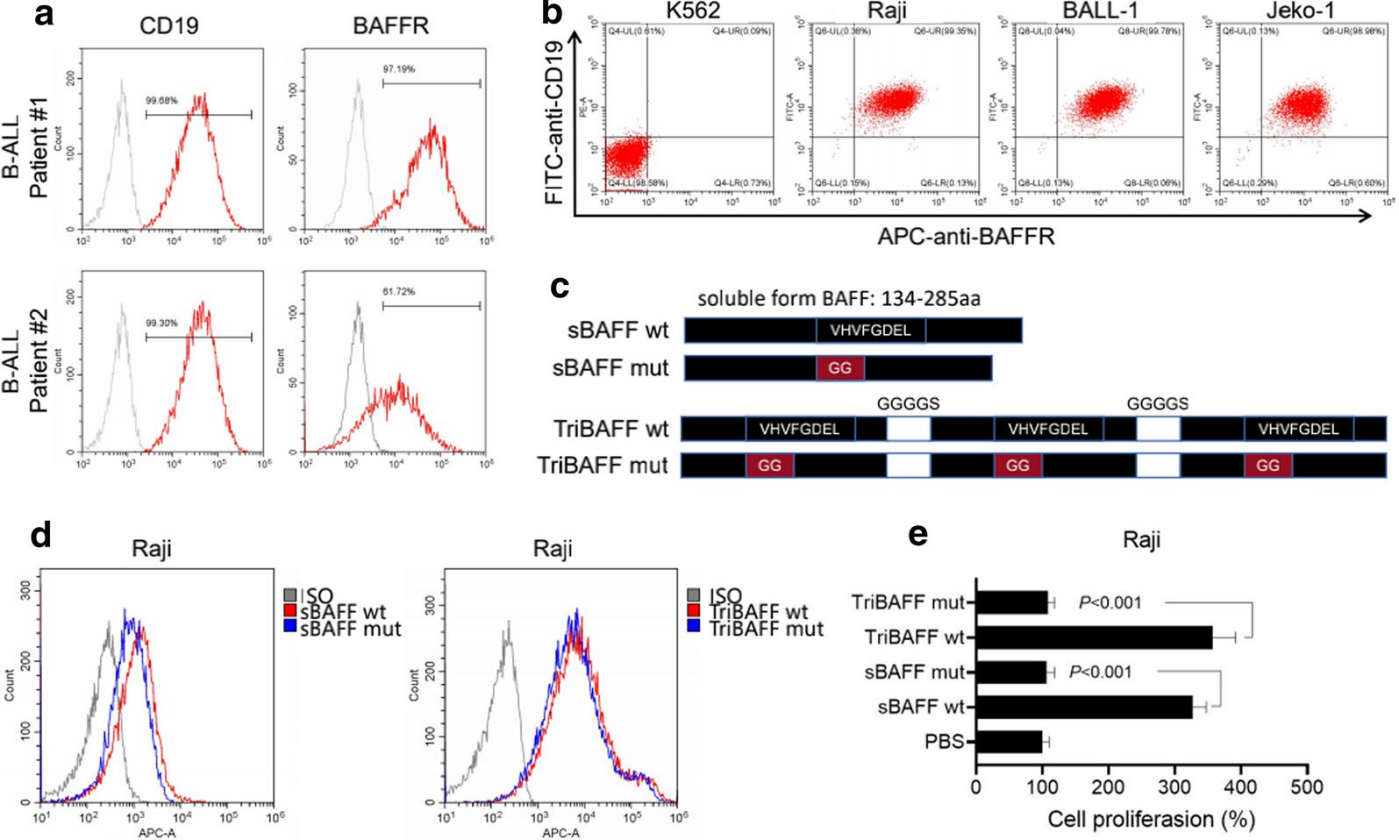
Mutated TriBAFF could bind but not activate BAFFR-bearing B cell lines. **a** PBMCs from two B-ALL patients were analyzed with CD19 and BAFFR using flow cytometry. **b** K562, Raji, BALL-1, and Jeko-1 were analyzed with CD19 and BAFFR using flow cytometry. **c** WT and mutated BAFF or TriBAFF were compared. **d** The binding affinity of WT and mutated BAFF or TriBAFF protein with Raji was detected using flow cytometry. **e** Proliferations of Raji were compared after adding WT and mutated BAFF or TriBAFF protein (5 µg/mL) into the culture medium

To study the expression of BAFFR, its presence on the surface of B cells from two patients with B-ALL and the B cell lines were analyzed using FACS. BAFFR was found to be expressed in approximately 99.30 and 61.72 % of B cells from the two patients, respectively (Fig. 1a). And according to flow results we found K562 did not express CD19 or BAFFR, the B cell lines of Raji, BALL-1, and Jeko-1 were all completely co-expressed with CD19 and BAFFR (Fig. 1b). To construct the vector binding but not activated BAFFR, the amino acids 217–224 (VHVFGDEL) of soluble BAFF were replaced by GG, and three mutated BAFFs were linked by the sequence of GGGGS to form the trimer as mutated TriBAFF (Fig. 1c). The recombinant WT and mutated BAFF, WT, and mutated TriBAFF could all bind to the surface of the Raji cells. Compared to the two recombinant BAFFS, the abilities of binding with Raji in two recombinant TriBAFFs were stronger (Fig. 1d), but the promotion of Raji proliferation was significantly inhibited by WT or mutated TriBAFFs (both P < 0.001; Fig. 1e).

### TriBAFF-CAR-T cells could cause lysis of BAFFR-bearing B cell lines in vitro

**Fig. 2:**
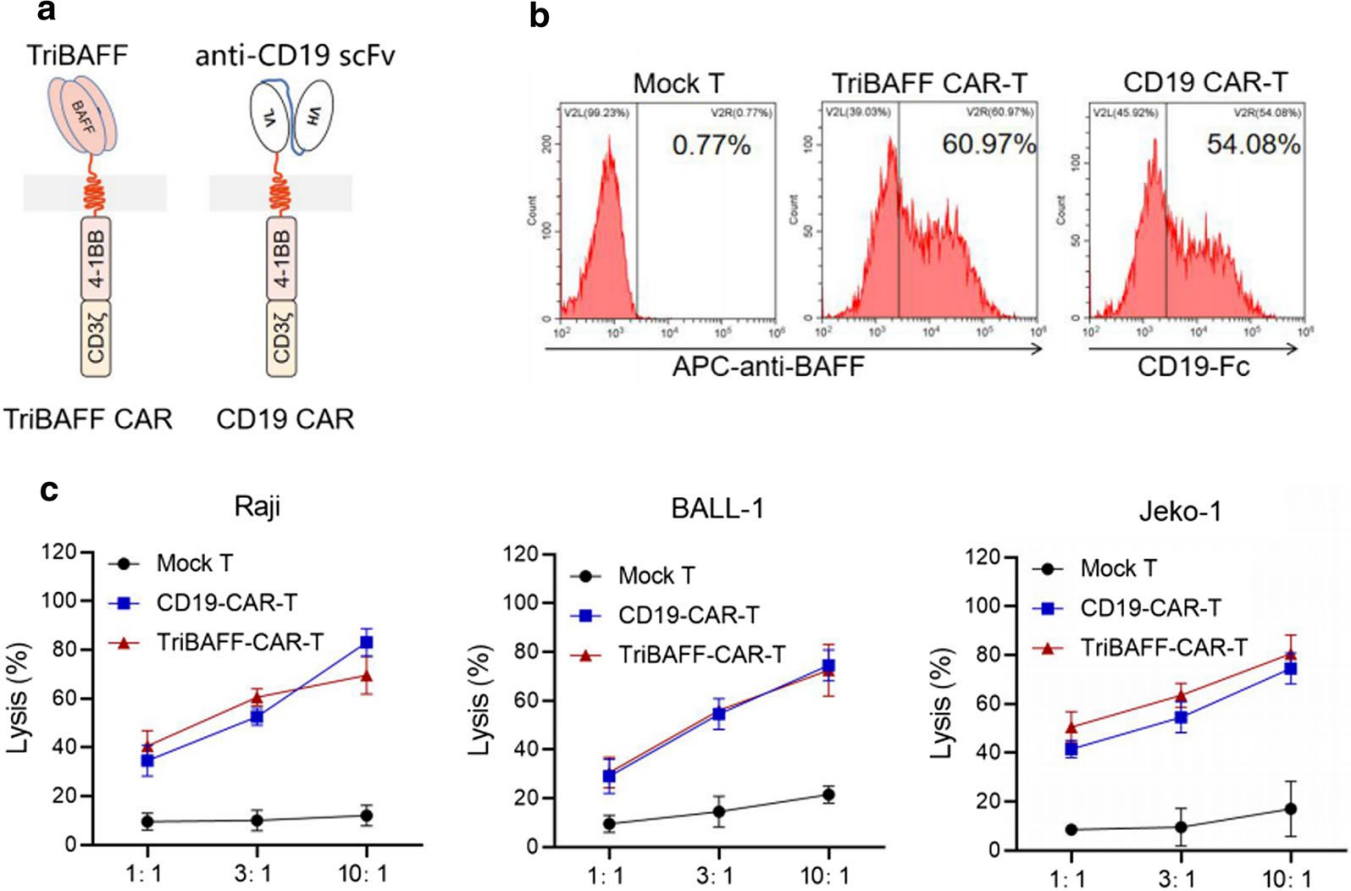
TriBAFF-CAR-T cells caused lysis of BAFFR-bearing B cell lines in vitro. **a** Structures of TriBAFF-CAR-T and CD19-CAR-T cells. **b** Efficacy of transfection was detected in TriBAFF-CAR-T and CD19-CAR-T cells. **c** When co-culturing with TriBAFF-CAR-T or CD19-CAR-T cells, the lysis of Raji, BALL-1, and Jeko-1 was analyzed at different effector/target ratios using the LDH assay

The TriBAFF-CAR was constructed by successively linking the signal peptide, mutated TriBAFF, hinge and transmembrane region of CD8α, costimulatory domain of 4-1BB, and signal transduction domain of CD3ζ. For CD19-CAR, the mutated TriBAFF was replaced by anti-CD19 scFv, which was designed from an anti-CD19 antibody (FMC63) and specifically recognized the antigen of CD19 (Fig. 2a). After the transfection of T cells by lentivirus packaging with TriBAFF-CAR or CD19-CAR, the efficacies of TriBAFF-CAR-T and CD19-CAR-T cells were found to be 60.97 and 54.08 %, respectively (Fig. 2b). When TriBAFF-CAR-T was co-cultured with Raji, BALL-1, or Jeko-1 (all were BAFF^+^ and CD19^+^), the proportion of lysed cells was comparable to the PC of CD19-CAR-T cells. As the effector:target ratio increased from 1:1, 3:1, and 10:1, the overall lethal effect gradually increased (Fig. 2c).

### TriBAFF-CAR-T cells could specifically diminished cell growth the BAFFR overexpressed cells in vitro

**Fig. 3:**
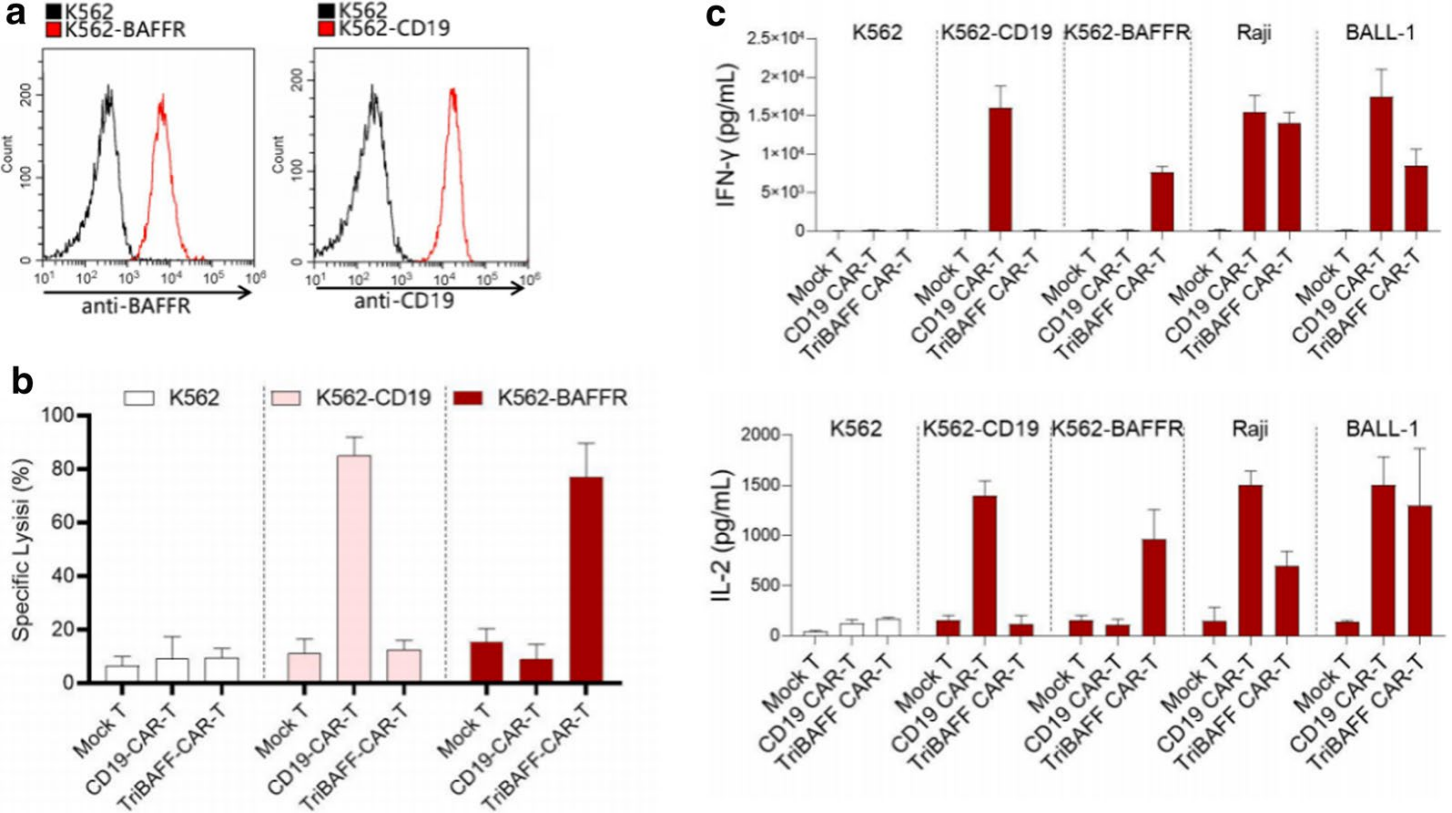
TriBAFF-CAR-T cells specifically killed the BAFFR overexpressed cells in vitro. **a **K562 cells were overexpressed with BAFFR and CD19. **b** Specific lysis of BAFFR- and CD19-bearing K562 was analyzed after coculturing with TriBAFF-CAR-T or CD19-CAR-T cells. **c** The levels of IFN-γ and IL-2 were detected in the supernatant of these cocultures

To test the specific inhibition effect of TriBAFF-CAR-T cells, we overexpressed BAFFR or CD19 in K562, which was naturally negative for BAFFR and CD19 (Fig. 3a). When the co-culture of BAFFR CAR-T or CD19-CAR-T cells with K562 was overexpressed by BAFFR or CD19, the specific lysis was elevated in the BAFFR CAR-T cell + BAFFR overexpressed K562 group and the CD19-CAR-T cell + CD19 overexpressed K562 group (Fig. 3b). In addition, the levels of IFN-γ and IL-2 secretion were largely increased in the two groups. Moreover, After treatment with BAFF-CAR-T or CD19-CAR-T cells in Raji or BALL-1cells the IFN-γ and IL-2 secretion were significantly increased to the same level as overexpressed K562 (Fig. 3c).

### TriBAFF-CAR-T cells inhibited the development of mouse B cell lymphoma in vivo

**Fig. 4:**
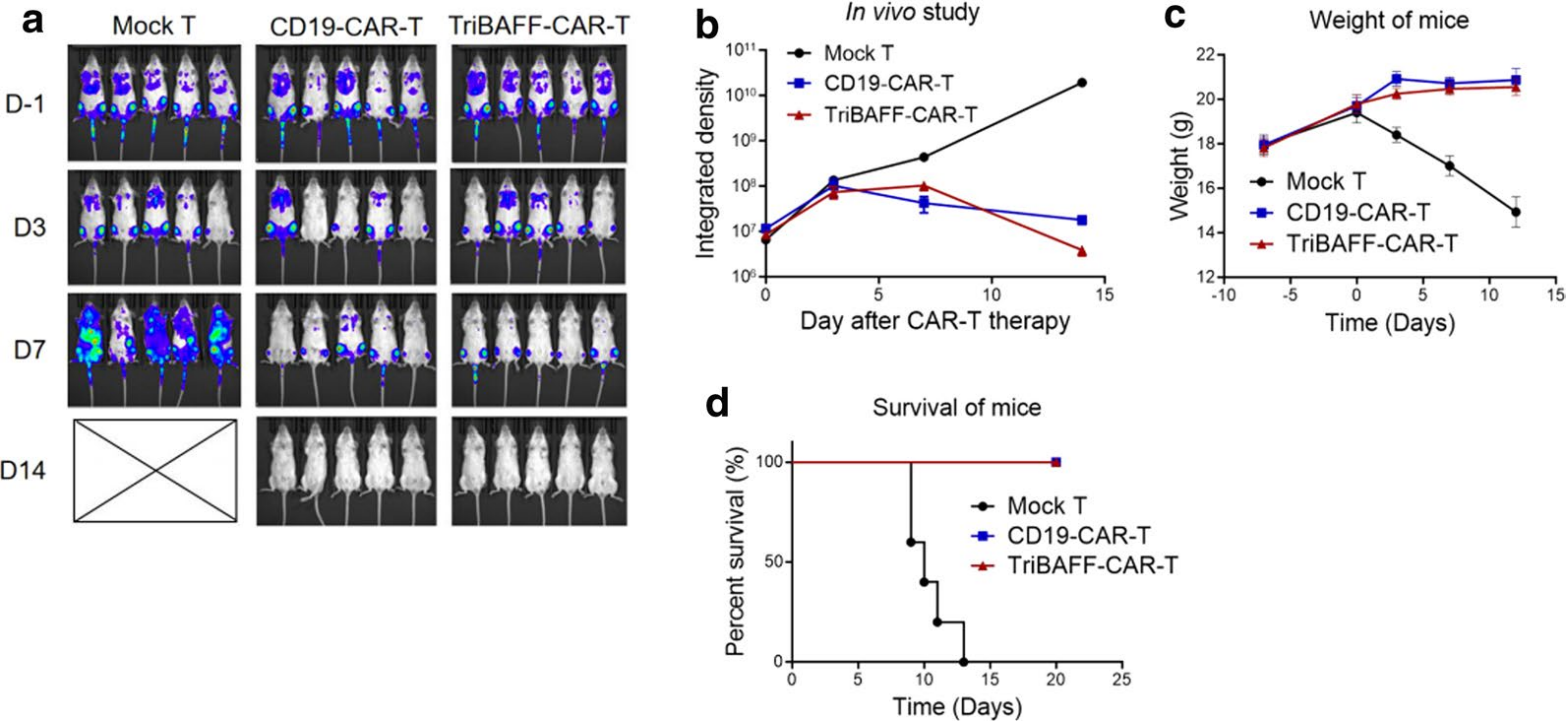
TriBAFF-CAR-T cells inhibited the development of mice B cell lymphoma in vivo. **a**Bioluminescence images, **b** integrated density of tumors, **c** weight of tumors, and **d** survival in Raji xenograft mice after administering mock T, CD19-CAR-T, and TriBAFF-CAR-T cells

The B-NDG mice were transfused with 1 × 10^9^/kg Raji cells to introduce B cell lymphoma. Mice in the mock group were found to have widespread proliferated Raji cells on day seven and finally died on day 14. However, TriBAFF-CAR-T and CD19-CAR-T cell therapy effectively inhibited the development of Raji cells, and the efficacies of the two CAR-T cell therapies were similar (Fig. 4a, b). Compared with the decreasing weight of the mock group, the trend of the TriBAFF-CAR-T and CD19-CAR-T cell groups was sustained at some elevations (Fig. 4c). All mice in the two CAR-T cell groups were still alive on day 20 (Fig. 4d).

### TriBAFF-CAR-T cells could inhibit the B cell line with CD19 knockout in vitro

**Fig. 5:**
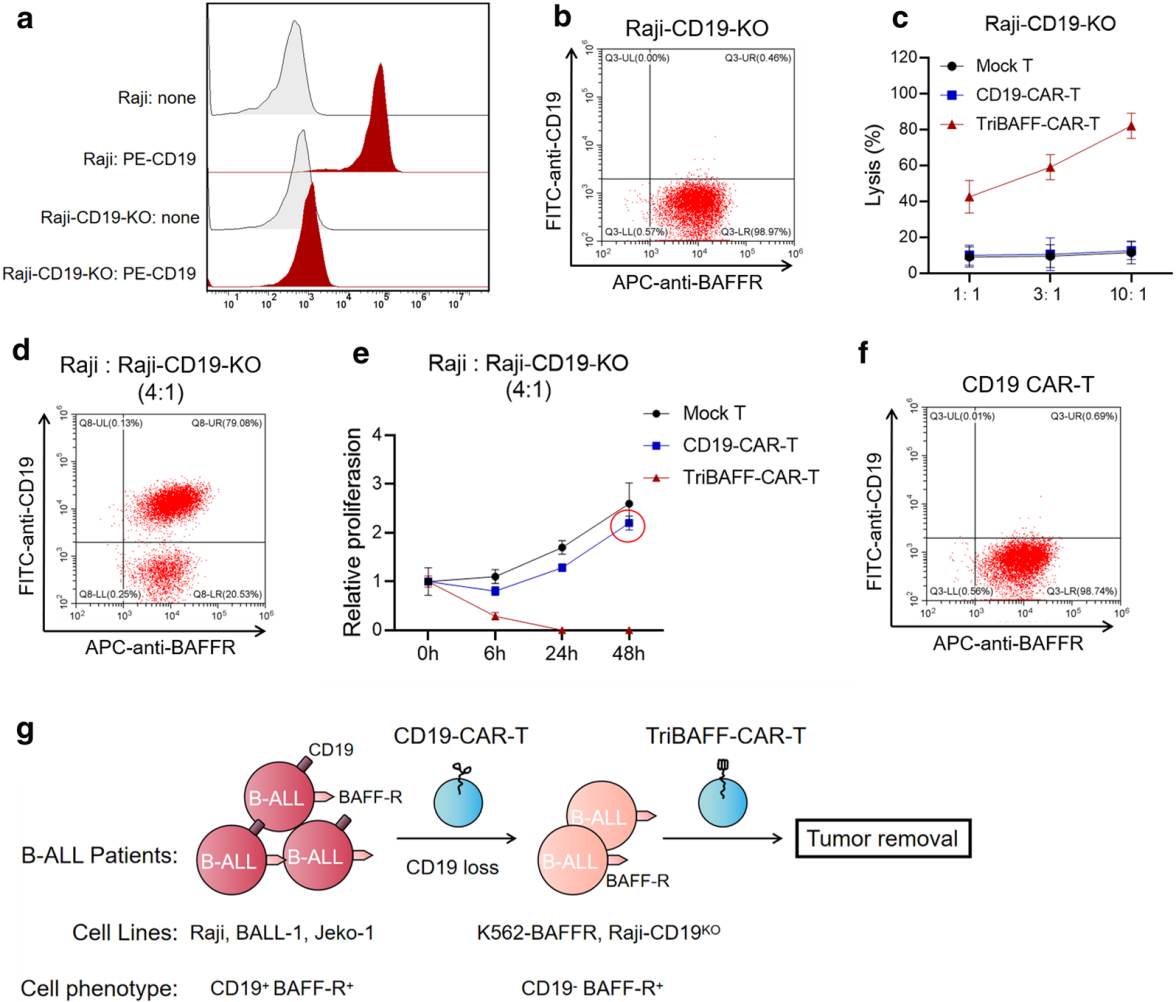
TriBAFF-CAR-T cells have the strong cytotoxicity to the B cell line with CD19 knockout in vitro. **a** Raji with CD19^KO^ was CD19-negative. **b** Raji with CD19^KO^ was still expressed in BAFFR. **c** Lysis of Raji with CD19^KO^ was detected after coculturing with TriBAFF-CAR-T or CD19-CAR-T cells at different effector: target ratios. **d** To stimulate the relapse BLL, Raji and Raji with CD19^KO^ were mixed at a ratio of 4:1. **e** The relative proliferation of mixed WT and CD19^KO^ Raji was detected after coculturing with TriBAFF-CAR-T or CD19-CAR-T cells for 48 h. **f** Flow cytometry indicated that mixed WT and CD19KO Raji were alive and BAFFR-positive after coculturing with CD19-CAR-T cells. **g** A flowchart for the research methodology

To verify whether TriBAFF-CAR-T is effective for CD19-CAR-T treatment of CD19 negative recurrence. Raji specifically knocked out of CD19 was detected as the model of negative for CD19 and positive for BAFFR (Fig. 5a, b). As the effector:target ratio increased from 1:1, 3:1, and 10:1, the lysis of Raji-CD19^KO^ caused by different doses of CD19-CAR-T cells was lower than that of TriBAFF-CAR-T cells. The lethal effects of CD19-CAR-T cells were the same as those transfected with the control (Fig. 5c). In addition, by combining the WT Raji with Raji-CD19^KO^ at a ratio of 4:1, TriBAFF-CAR-T cells effectively inhibited the two types of Raji, while CD19-CAR-T cells only killed the WT Raji with CD19^+^, and the Raji with CD19^−^ continued to proliferate after subsequent co-culture. FACS results further demonstrated that live CD19 Raji cells still expressed BAFFR, indicating that TriBAFF-CAR-T cells exhibited a lethal effect by targeting BAFFR (Fig. 5e, f).

## Discussion

CD19-CAR-T cell therapy has been demonstrated to have therapeutic efficacy in patients with B cell malignancies, such as B-ALL, relapse, and refractory lymphoma [29]. Alternations of the dominant marker account for approximately 30 % of resistance after CAR-T cell therapy,including antigen loss and antigen-positive recurrence, so a novel target is urgently needed for these relapses, such the combination of other chemical drug [30, 31] or find other new targets CAR-T [9]. Previous studies have revealed that CAR T cells targeting BAFF-R can overcome CD19 antigen loss in B cell malignancies [32].In this study, we found a new methods to inhibited the BAFFR-positive cell viability by constructing a TriBAFF directing CAR-T cells and further demonstrated their ability to inhibit BAFFR-positive cells both *in vitro* and *in vivo.* Lastly, in the CD19^KO^ B relapse lymphoma cell line model, when CD19-CAR-T cells lost their efficacy, the TriBAFF-CAR-T cells caused the lysis of B cell lines.

Some studies have revealed that therapy targeting BAFF for autoimmune diseases might worsen a patient’s condition. As T cells are the main regulators of autoimmune diseases, the hypothesis of T cell exhaustion conducted by BAFF may be correct [33]. In addition, some cases of B cell malignancies have shown that BAFF concentration is elevated in tumor microenvironments and cells, thus, the role of BAFF on B cells and other lymphocyte or myeloid cells is the opposite [34]. The different effects on non-B cells of BAFF could be mediated by receptors of TACI and BCMA. In the future, if TriBAFF-CAR-T cells are administered to patients, the adverse effects on T cells or other myeloid cells should be considered [32] .

In addition to the target of CD19, other B-lineage markers directing CAR-T cells, such as CD22-CAR-T cells, display considerable potency and have shown efficacy comparable to that of CD19-CAR-T cells [35]. However, the phenomenon of relapse associated with CD22 diminishing in lymphoma blasts is similar to CD19 antigen loss [36]. CD22-negative cells escape killing by CD22-CAR-T cells. In addition, CD20-CAR-T cells conduct CD20-positive specific lysis and cytokine secretion, even in CD20-downregulated lymphoma and leukemia [37].

This second-generation CAR-T cell type consists of scFv from a ligand fused to the signaling transductor of CD3 and costimulatory sequence of CD28 or 4-1BB. This structure benefits its capacity against tumors with a deficiency of HLA [38, 39] and targets the receptor-bearing cells with full activation signals. In addition, the binding affinity between BAFF and BAFFR is important for CAR-T cell activation, which influences the killing of tumor cells. TriBAFF, under normal physiologic conditions, is soluble [40] and can better produce and express on the surface of T cells. Moreover, although the binding abilities of BAFF for its three receptors have been described, the binding affinity between different forms of BAFF and BAFFR has not yet been well investigated [41, 42]. Nicoletti et al.. determined the IC_50_ and EC_50_ values of an antagonist inhibiting the linkage of BAFFR to TriBAFF or membrane-bound BAFF, finding that TriBAFF is more potent than membrane-bound BAFF in stimulating BAFFR-mediated NF-kB activity [28].

Given the lack of a murine model of relapse lymphoma after attacking pressure of CD19-CAR-T cells [32], the CD19^KO^ Raji cell line was detected as negative with CD19 but positive with BAFFR. However, the expression level of BAFFR in the CD19^KO^ Raji cell line was not elevated; thus, the potential binding with BAFF-CAR-T cells may not be strengthened.

Our study had some limitations. (1) A xenograft B-ALL model was used to test the efficacy of CAR-T cells, which is not a perfect stand-in for this disease. For future studies, another PDX model should be considered. (2) Although the weights of mice after CAR-T cell therapy were nearly retained at baseline levels, the xenograft B-ALL model could not comprehensively evaluate the toxicity and safety of TriBAFF-CAR-T cells. Therefore, a more suitable model is needed. (3) The immunodeficient mice used in this study did not reflect the influence of TriBAFF-CAR-T cells on the immune system. Inducing B-ALL disease in mice may better show the efficacy of TriBAFF-CAR-T cells. (4) TriBAFF-CAR-T cell therapy for the recurrence of B-ALL with CD19 antigen loss was studied based on cell lines. Another PDX model or induced recurrence of B-ALL could provide further insights.

## Conclusions

In this study, we successfully constructed a novel TriBAFF-CAR-T cell to eliminate BAFFR-bearing and CD19 antigen loss hematological tumor cells both *in vitro* and in vivo (Fig. 5g).

## Data Availability

The datasets used and analyzed in the current study are available from the corresponding author in response to reasonable requests.

## Abbreviations

ALL: Acute lymphoblastic leukemia
APC: Antigen presenting cells
BAFFR: BAFF receptor
BAFF: B cell activating factor
BCMA: B cell maturation antigen
CD19^KO^: CD19 knockout
CAR-T cell: Chimeric antigen receptor T cell
TACI: Cyclophilin ligand interactor
DMEM: Dulbecco’s modified Eagle’s medium
IFN-γ: Interferon gamma
IL: Interleukin
NC: Negative control
PBMC: Peripheral blood mononuclear cell
PC: Positive control
scFv: Single-chain variable fragment
TriBAFF: Trimer of BAFF
WT: Wild-type
